# Multiparametric quantification of bacterial cells using digital holographic microscopy

**DOI:** 10.1038/s41598-025-24917-5

**Published:** 2025-11-20

**Authors:** Álvaro Cano, Adrián Sanz-Jiménez, Oscar Malvar, Jose Jaime Ruz, Priscila M. Kosaka, Sergio García-López, Montserrat Calleja, Javier Tamayo

**Affiliations:** Instituto de Micro y Nanotecnología, IMN-CNM (CEI UAM + CSIC), Isaac Newton 8, Tres Cantos, Madrid, 28760 Spain

**Keywords:** Digital holographic microscopy, Bacterial cells, Nanomechanical mass spectrometry, Dry mass quantification, Biological techniques, Biophysics, Microbiology, Optics and photonics

## Abstract

The detection and quantification of the physical properties of bacterial cells are crucial for advancing precision microbiology. Here, we use digital holographic microscopy (DHM) to measure the dry mass of individual *Staphylococcus epidermidis* (*S. epidermidis*) and *Escherichia coli* (*E. coli*) cells with high throughput and rapid acquisition. We demonstrate that by processing quantitative phase images with polynomial background correction, Gaussian filtering, and adaptive masking, we are able to obtain, not only the dry mass of individual bacterial cells, but also detailed morphological information. These features allow the discrimination between single and clustered cocci, as well as the identification of elongation patterns in bacilli, that may provide relevant indicators of bacterial growth and physiological state. Finally, we compare the DHM dry mass distribution with those obtained by nanomechanical mass spectrometry (NMS), highlighting the potential and limitations of each technique.

## Introduction

Real-time and high-throughput classification of bacterial cells is critically important in a wide range of applications, from food pathogen detection, biomedical research, clinical diagnostics to antimicrobial resistance monitoring. Among these, bacterial infections are of particular concern, as they can rapidly progress into sepsis^1^, leading to around 49 million cases and 11 million deaths per year worldwide^2^. On the other hand, the indiscriminate overuse and misuse of antibiotics has accelerated the emergence of super-resistant bacterial strains^3,4^. Recently, the World Health Organization declared antimicrobial resistance as one of the 10 public health challenges facing the humanity. In this context, there is an urgent need for rapid, simple and innovative strategies for bacterial identification and classification.

The dry mass of bacteria, defined as the mass of the non-aqueous cellular constituents, such as proteins, nucleic acids, carbohydrates and lipids, has gained a lot of attention in the last decade^5–7^. Dry mass, together with morphological features, serves as one of the most useful indicators of bacterial physiology. Since proteins play a central role in cellular function and account for roughly half of the total dry mass^8^, its measurement provides valuable insight into the biosynthetic and degradative processes within the cell.

Several technologies have recently emerged for measuring biophysical properties of bacterial cells^9^. For instance, electron microscopy techniques^10,11^ offers unprecedented spatial resolution to characterize morphological properties of bacterial cells, but it requires complex sample preparation, it operates under vacuum conditions and it does not provide quantitative information on dry mass. Optical microscopy, although is widely accessible in most laboratories, suffers from low contrast, due to most of cells being transparent, and lacks quantitative capabilities. However, transparent biological samples can generally interact with light inducing changes in its phase. This phenomenon led to the development of optical-contrast enhancing imaging techniques. For instance, phase contrast, proposed by Zernike^12^, and differential interference contrast^13^, significantly enhance contrast and allow imaging of transparent samples; however, these techniques still cannot quantify the dry mass.

To overcome these limitations, different biophysical methods have recently emerged. Suspended microchannel resonators^14–17^ (SMRs) and nanomechanical mass spectrometry (NMS)^7,18–22^ offer femtogram resolution in mass measurement of single cells. A SMR measures the buoyant mass of suspended cells as they flow through a hollow resonator, requiring the same bacterial cell to be measured in two fluids of different densities, which is technically complex and limits the throughput. In NMS, the cells are individually adsorbed onto the surface of a nanomechanical resonator, producing a measurable downshift in the resonance frequencies. These frequency shifts enable the extraction of the mass of each cell with sub-femtogram resolution. However, NMS typically operates under vacuum conditions and requires complex instrumentation, limiting the throughput and making these technologies less suitable for real-time applications.

In this context, quantitative phase imaging (QPI) has gained considerable attention over the last decades as a label-free, non-invasive and real-time technique for biological applications^23–27^. By analyzing optical phase shifts, QPI provides quantitative maps of cellular dry mass, refractive index, and morphology, offering insights into cellular physiology. Recent advances in QPI have enabled single-bacterium resolution^28–32^ while maintaining high-throughput capabilities, making it suitable for population-level studies. Moreover, QPI has been successfully employed to quantitatively characterize cellular dynamics, biophysical properties, and responses to external stimuli^6,33,34^.

DHM^35–38^, a subclass of QPI, is emerging as a promising tool for the identification and classification of cells. In this work, we employ a transmission DHM to measure the dry mass and morphological features of two representative bacterial species, *Staphylococcus epidermidis* and *Escherichia coli*. We developed a dedicated image processing workflow, including a polynomial background correction, Gaussian filtering, and adaptive masking, to extract, not only the dry mass of individual cells but also morphological information. This multiparametric approach enables a richer characterization of bacterial cells and supports classifications strategies beyond single-parameter analysis. These parameters allow us to distinguish between single and clustered cocci (spherical bacterial cells) and to identify elongation trends in bacilli (rod-shaped bacterial cells). We also applied the Koch & Schaechter model^39,40^, which relates the size distribution of steady-state populations of asynchronously growing cells to the mean mass at the instant of division. Finally, we compare the dry mass distributions and mean division mass obtained by DHM with those measured using NMS, validating our approach against a well-established technique.

## Results and discussion

### Experimental setup

**Fig. 1:**
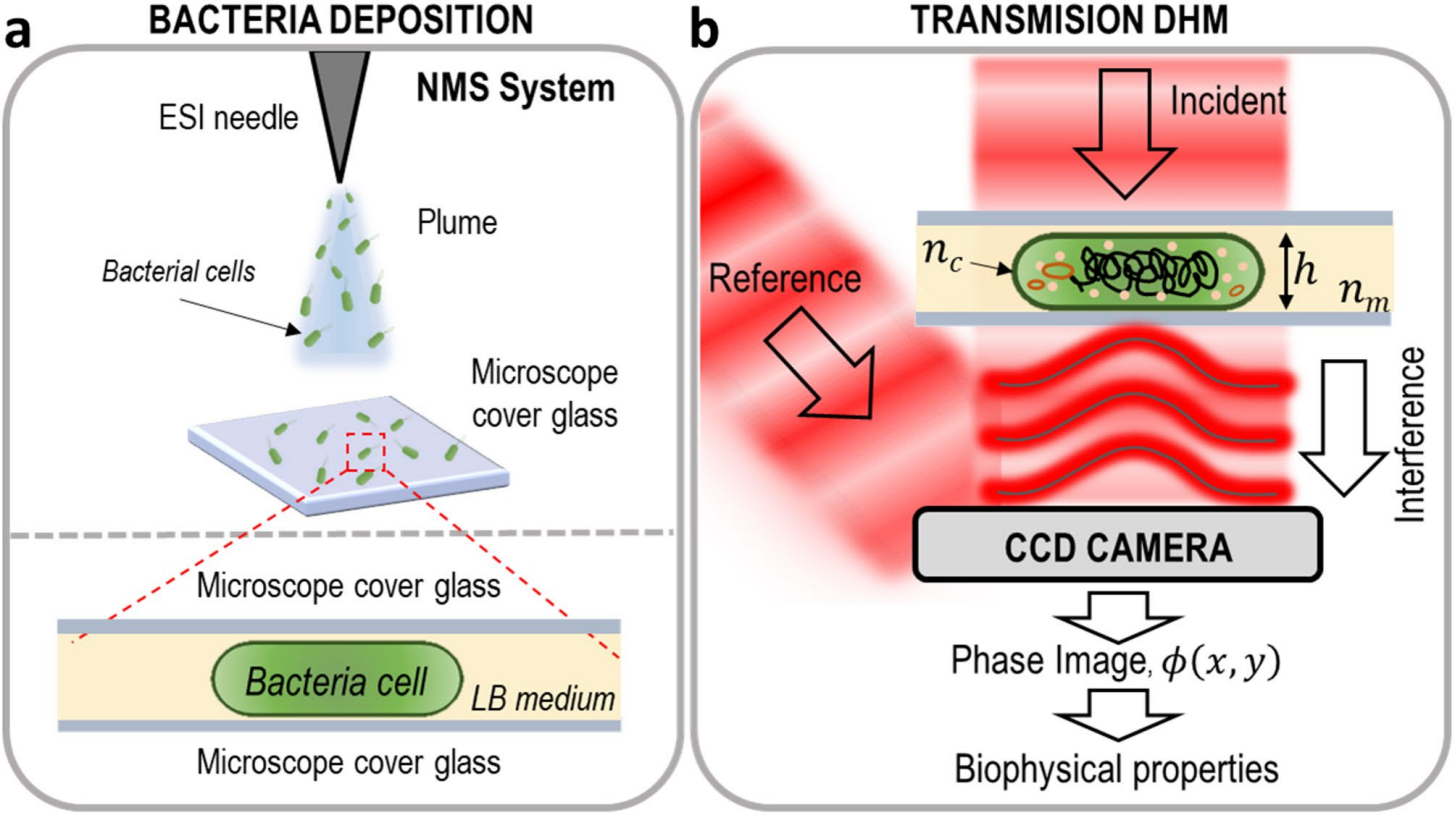
Schematic of bacterial deposition and quantitative transmission DHM. (**a**) Schematic representation of the deposition of bacterial cells on a microscope cover glass using electrospray ionization technique (top). After nebulization, bacterial cells are sandwiched between two cover glasses with a LB medium drop (bottom). (**b**) Transmission digital holographic microscopy setup. A laser beam is split into a reference and an incident beam that illuminates the sample. The off-axis interference of both wavefronts produces holograms recorded by a CCD camera. After digital processing of the images, the biophysical properties of the cells are extracted.

Bacterial cells were nebulized for 30 min by electrospray ionization technique^7,22^ (ESI) onto a microscope cover glass, using a previously described nanomechanical mass spectrometry prototype^7^ operating under vacuum conditions (Fig. 1a, top), ensuring a homogeneous distribution of bacterial cells on the surface and minimizing cell aggregation. After the nebulization process, a drop of LB medium was added to the cover glass and a second one was placed on top, sandwiching the bacterial cells between the two surfaces^28^ (Fig. 1a, bottom). The deposition protocol inactivated bacterial cells while preserving their overall structure. Then, DHM measurements were performed after a short stabilization period to reduce sample drift.

Phase images were acquired using a commercial DHM (DHM - T-2100, Lyncée tec) in transmission configuration^6^. The transmitted object wavefront and the reference beam were combined off-axis to create a spatially modulated interference pattern, known as hologram, which was digitally recorded by a charge-coupled device (CCD) camera (Fig. 1b). Each hologram was acquired in 1 s (20 frames at 0.05 s exposure), which, together with bacterial inactivation, prevented significant particle motion or medium-induced changes during acquisition^32^.

As detailed above, the DHM images of the bacterial cells were carried out in LB medium. In these conditions, the phase shift ($\:\varphi\:\left(x,y\right)$) at each point of the cell is proportional to the difference between the refractive index of the cell ($\:{n}_{c}\left(z\right)$), the refractive index of the medium ($\:{n}_{m}$) and the cell height ($\:h$) given by ^24,26,38^,

1 $$\:\varphi\:\left(x,y\right)=\frac{2\pi\:}{\lambda\:}{\int\:}_{0}^{h}\left({n}_{c}\left(z\right)-{n}_{m}\right){dz}\:$$

The refractive index difference, $\:{n}_{c}\left(z\right)-{n}_{m}$, is proportional to the mass density of the non-aqueous constituents of the bacterial cells. Given the equivalent variation of the optical path difference (OPD) that can be expressed as,

2 $$\:{OPD}\left(x,y\right)=\:\frac{\lambda\:}{2\pi\:}\varphi\:\left(x,y\right)$$

and considering that the proportionality constant for most of the biomolecules that make up the cell falls within a narrow range^41,42^, the phase inside the regions that contain bacteria is directly related to the dry mass surface density ($\:\sigma\:\left(x,y\right)$) by the following relationship,

3 $$\:\sigma\:\left(x,y\right)=\:\frac{\lambda\:}{2{\pi\:\alpha\:}}\varphi\:\left(x,y\right)=\frac{{OPD\:}(x,y)}{\alpha\:}\:$$

where $\:\alpha\:$ is the refractive index increment, with a value of $\:1.9\times\:{10}^{-4}\:{m}^{3}/kg$ according to Zhao et al.^41^, who analyzed thousands of proteins and reported a very narrow distribution. This relationship enables the quantitative and label-free characterization of the dry mass of bacterial cells, $\:{M}_{i}$, from the phase measurement given by:

4 $$\:{M}_{i}=\frac{1}{\alpha\:}{\iint\:}_{{S}_{i}}^{\:}{OPD}\left(x,y\right){dxdy}=\frac{{S}_{i}}{\alpha\:}\stackrel{-}{{{OPD}}_{i}}\:$$

where $\:{S}_{i}$ is the bacteria area, and $\:\stackrel{-}{{OPD}_{i}}$ is the mean OPD of the region that contain the bacteria $\:i$.

### Phase background leveling, filtering and adaptive masking

#### Background leveling

Accurate characterization of the dry mass and morphological parameters of bacterial cells using DHM requires precise estimation of the background noise in order to remove it across the field of view. This background correction is necessary to significantly improve the precision of the phase measurement and, finally, the correct quantification of the bacterial cells’ dry mass. First, we calculated the OPD from the integrated phase images (Eq. 2). Then, we applied a 2D polynomial surface fitting^43^ to the OPD images to compensate undesirable background curvature and tilt. This step removes low-frequency artifacts and corrects uneven illumination in the phase images.

**Fig. 2:**
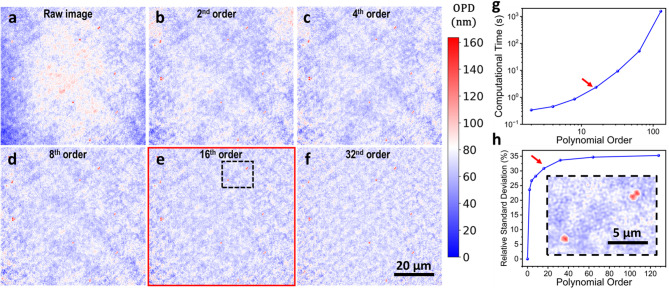
Background leveling. (**a**) Raw optical path difference (OPD) before background correction. (**b**–**f**) Background leveled OPD images after applying a polynomial fitting of order 2, 4, 8, 16 and 32, respectively. Higher polynomial orders progressively reduce undesirable background curvatures. (**g**) Graph of the computational time in seconds as a function of the polynomial order. Increasing the polynomial order dramatically increase the computational time. (**h**) OPD standard deviation, relative to the raw image, as a function of the polynomial order. A polynomial order of 16th achieves a $\:\sim\:30\%\:$reduction in low-frequency noise. The inset shows a zoomed region of bacterial cells highlighted in (**e**). Red arrows indicate the selected polynomial order (16th ) as a good trade-off between computational time and noise reduction, without compromising bacterial cells information.

In order to quantify how the polynomial order influences background leveling, we computed the OPD standard deviation over the entire image as a function of the polynomial order. Here, the whole image can be used considering the big difference in size of the image and the bacterial cells. Additionally, due to the importance of computational efficiency for implementing real-time and high-throughput analysis, we performed a benchmark study in a DHM image with a size of ($\:802\:\times\:\:764$) pixels, that corresponds to a total area of around $\:5\times\:{10}^{3}\:{\mu\:m}^{2}$. Fig. 2a shows the OPD raw image of *S. epidermidis* deposited and sandwiched between two microscope cover glasses before applying the background correction, where an uneven illumination is clearly visible. Figs. 2b-f show polynomial fits of orders 2, 4, 8, 16, and 32 that were applied to the OPD of the image, respectively. As shown in Figs. 2a-f, increasing the polynomial order improves background correction by reducing low-frequency noise. In Fig. 2h, we represent the relative standard deviation reduction with respect to the raw image. A polynomial of order 16 reduces the low-frequency noise by approximately 30% while requiring only around 1 s of computational time (see Fig. 2g) on a standard desktop computer (*13th Gen Inter® Core™ i7-13700 F 2.10G GHz*,* 64 GB of RAM*). In contrast, higher-polynomial orders provided negligible additional noise reduction but increase significantly the computational time. Therefore, we selected a polynomial order of 16 (highlighted with red arrows in Figs. 2g and h) as an optimal trade-off between computational cost and background noise suppression. As shown in the zoomed region (inset of Fig. 2h) this correction does not introduce any shape artifacts in bacterial cells images, preserving their structural shape.

#### Gaussian filtering

After performing background leveling using a 16th order polynomial fitting, we applied a Gaussian filter to the corrected OPD images to reduce the high-frequency noise. The Gaussian filter works by convolving the image using a Gaussian kernel (a matrix whose values follow a normal distribution centered on each pixel). The size of the kernel is the user-defined radius, which corresponds to the standard deviation ($\:\sigma\:=r$) of the Gaussian function, $\:\text{G}\text{F}=\:\frac{1}{2\pi\:{\sigma\:}^{2}}{e}^{-\frac{{x}^{2}+{y}^{2}}{2{\sigma\:}^{2}}}$, and controls the degree of smoothing. This filter improves the smoothness of the OPD profile of the bacterial cells, without modifying the morphology, and will help to correctly perform the subsequent adaptive masking procedure step.

**Fig. 3:**
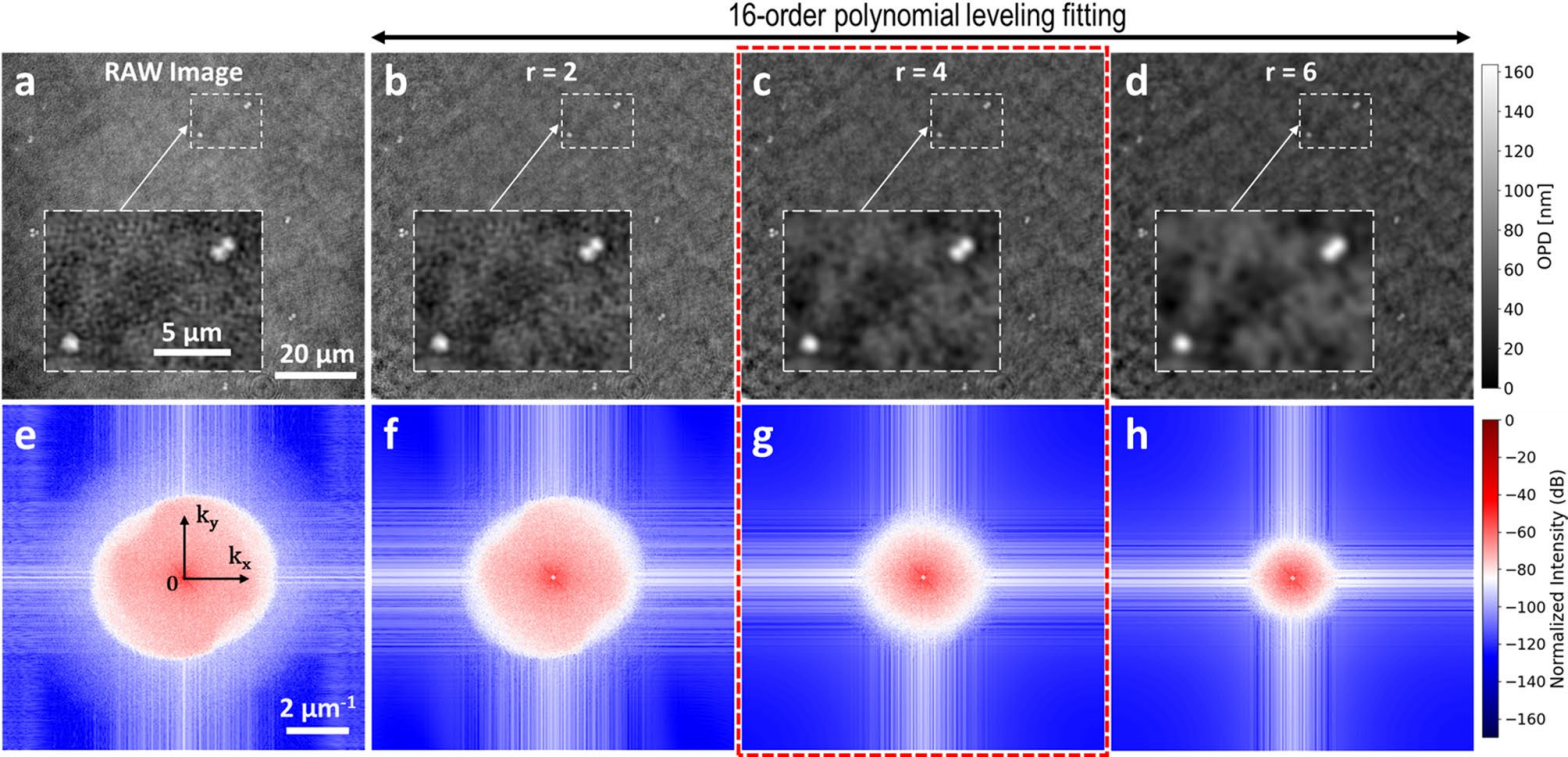
Gaussian filtering. (**a**) Raw optical path difference (OPD) before background leveling and Gaussian filtering. (**b**–**d**) OPD background leveled images after 16th order polynomial fitting and Gaussian filtering with radii *r* = 2, 4 and 6, respectively. Insets show the smoothing effect of bacterial cells. (**e**–**h**). Normalized intensity in logarithmic scale (dB) of the 2D discrete Fourier transform of images (**a**–**d**), respectively. 2D-DFT of the raw image (**e**) shows low-frequency components near the center, which are reduced by the background leveling step. Increasing the radii of the Gaussian filter reduce the high-frequency noise components. We have selected a radius of 4 as the optimal tradeoff between noise reduction and preservation of critical bacterial information (red dashed box). Axes k_x_ and k_y_ represent spatial frequencies in µm^− 1^, given by the physical pixel size (~ 90.6 nm).

To select the better radius for the high-frequency filtering step, we performed a parametric study applying different kernel radii to the leveled image and then we analyzed the results in the frequency domain via a 2D Discrete Fourier Transform^44,45^ (DFT). The goal is to find the best trade-off between removing the high-frequency noise and the conservation of the shape and intensity of the bacterial signal. In the Fourier plane, low-frequencies components are concentrated near the center and correspond to smooth background variation, while high-frequencies are distributed towards the edge and corresponds to fine details. Fig. 3a shows the original raw image (before polynomial correction and Gaussian filter) and Figs. 3b-d show the background leveled images after applying the 16th order polynomial correction followed by Gaussian filtering with kernel radii of 2, 4 and 6, respectively. As shown in the insets of Figs. 3b-d, increasing the filter radius progressively smooths the shapes of the bacterial cells. Figs. 3e-h show the corresponding 2D-DFT normalized magnitude spectra, using logarithmic scale in decibels (dB) to enhance the contrast. The 2D-DFT of the raw image (Fig. 3e) exhibits low-frequency components near the center, which are attenuated by the polynomial leveling (Figs. 3f-h). As shown in Fig. 3f, a radius of 2 preserves the high-frequency noise, while a radius of 4 (Fig. 3g) significantly reduces the noise without compromising the shape or intensity information of the bacterial signal. Applying a filter with larger radius leads to over-smoothing and a significantly distortion of the cell’s morphology (see Figs. 3d and h). In this work, we selected a radius of 4 (highlighted with a red dashed box in Fig. 3) as the optimal trade-off between noise reduction and preservation of bacterial information.

#### Adaptive masking

The third step to characterize the bacterial cells properties was to isolate them from the background noise. There exist different types of segmentation algorithms^46–48^. Here, after applying the 16th order polynomial fitting and the Gaussian filtering, we applied an adaptive binary masking procedure to the OPD images. The threshold was computed using a Gaussian adaptive method with a block size of 127 pixels and an offset defined as a multiple of the image’s standard deviation, optimized to preserve bacterial features. The resulting binary mask was refined using a morphological opening step with a disk-shaped element with a user-defined radius. This performs an erosion followed by a dilation, that removes small objects and noise, smooths the object contour and preserves the shape of the cells. Figs. 4a and e show these binary masks optimized for spherical shapes such as *S. epidermidis* and for rod-like shapes like *E. coli*. Subsequently, labeled regions were further dilated using a disk-shaped element. This final step helps to connect nearby objects, fill smalls holes and expand segmented regions, effectively correcting fragmented or incomplete segmentations. Figs. 4b and f show the resulting labeled images, where well-defined regions corresponding to bacterial cells are observed.

Figs. 4c and 4 g show OPD cross-section extracted along the white dashed lines in the inset images, zoomed from Figs. 4b and f (yellow boxes), respectively. The dark dashed line represents the mean of the OPD background noise of each image, while the orange shaded area represents the noise level defined as the $\:mean\left(OPD\right)\pm\:\:std\left(OPD\right)$, where $\:std\left(OPD\right)$ represents the OPD standard deviation. This will later be used to estimate the dry mass distribution of bacterial cells. Figs. 4d and h represent a 3D OPD image of a *S. epidermidis* and *E. coli*, respectively, where the remaining background noise, after the leveling and Gaussian filtering procedure, is clearly visible.

**Figure:**
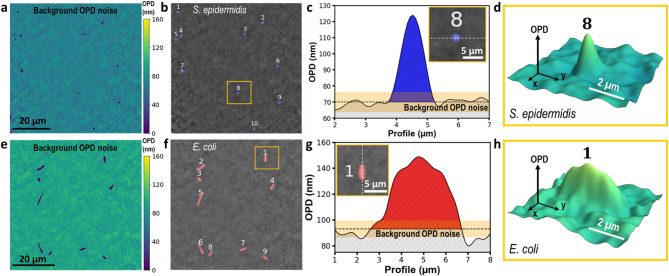
Fig. 4 Adaptive masking for segmenting *S. epidermidis* and *E. coli* cells. (**a**,**e**) Masks obtained after applying the adaptive thresholding and morphological opening optimized for *S. epidermidis* and *E. coli*, respectively. (**b**,**f**) Labeled cells extracted from the morphological dilation, where the segmented bacteria are clearly visible. (**c**,**g**) OPD cross-sections extracted along the white dashed lines in inset images (zoomed views of **b** and **f**, yellow boxes). Dark-dashed lines represent the mean background extracted from (**a**) and (**e**), and the orange shaded region indicates the OPD noise level. (**d**,**h**) Representative 3D OPD image of the *S. epidermidis* and *E. coli* extracted from the yellow boxes in inset images f and g, respectively. Both images use the same color bar scale as in **a** and **e**.

#### Characterization of the dry mass and morphological parameters of bacterial cells

After applying the polynomial fitting and Gaussian filtering, region properties such as area, perimeter, eccentricity, circularity, and major/minor axis lengths, among others, were extracted from these labeled objects. The individual dry mass of each cell was calculated by integrating the total OPD within each labeled region (Eq. 4).

**Fig. 5:**
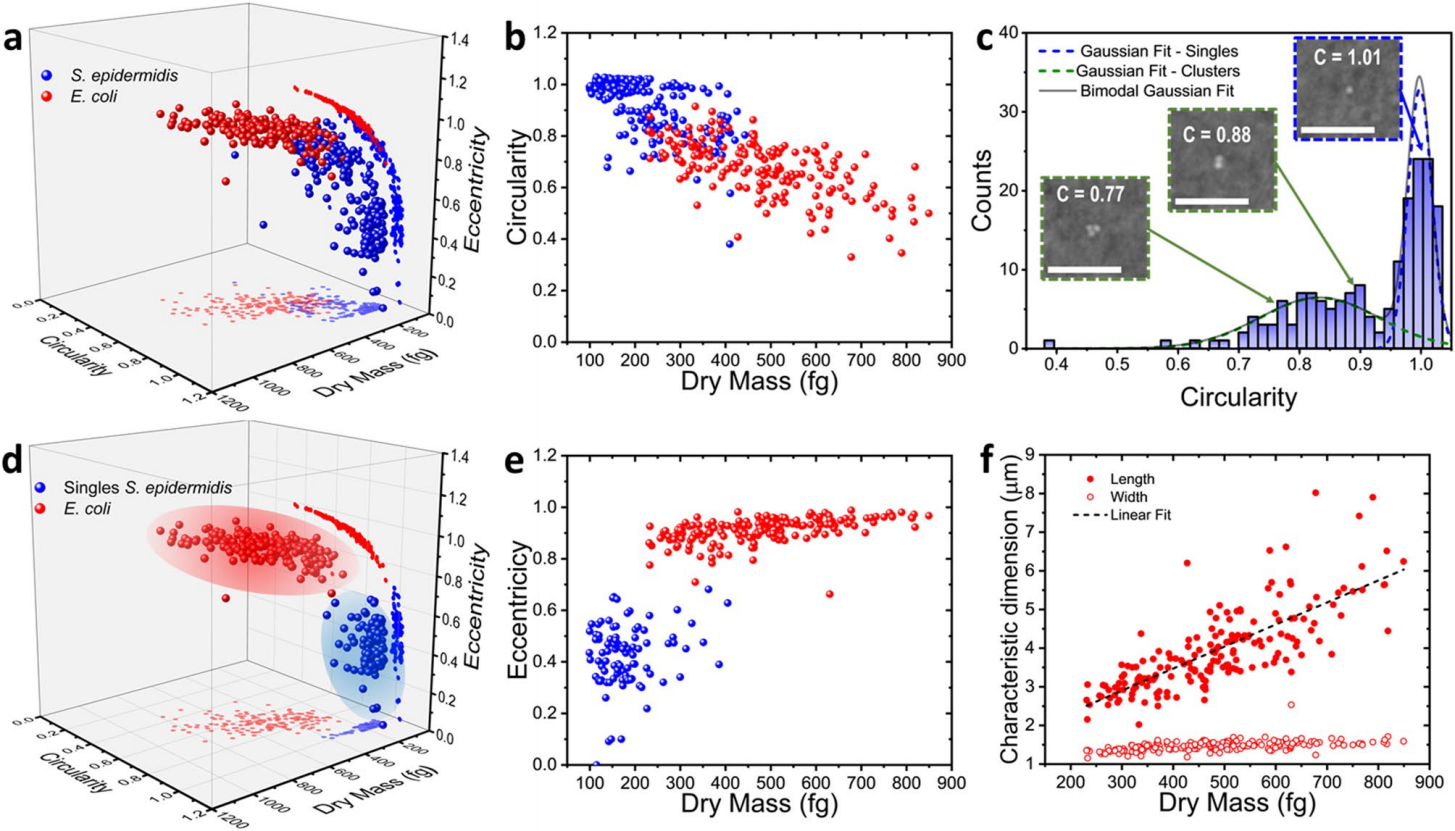
Morphological and dry mass characterization of bacterial cells. (**a**) 3D scatter plot of dry mass versus circularity and eccentricity for all labeled regions corresponding to *S. epidermidis* (blue dots) and *E. colis* (red dots). (**b**) Circularity versus dry mass, showing morphological differences between both strains. (**c**) Histogram of circularity for *S. epidermidis*, fitted with a GMM model. The total fit is shown as a solid gray line, the single cells as a dashed blue line and clusters as dashed green line. This model is used to filter single and clustered cells. (**d**) 3D scatter plot after applying GMM-based filter to include only single *S. epidermidis* cells, where two populations are clearly visible. (**e**) Eccentricity versus dry mass for single *S. epidermidis* and *E. coli*. While *E. coli* show values close to 1, *S. epidermidis* displays a wider distribution. (**f**) Length (red filled circles) and width (red open circles) versus dry mass for *E. coli*. While the width is almost constant, the length linearly increases with the dry mass.

From a total of 29 and 27 DHM images, we obtained 179 and 177 labeled regions for *S. epidermidis* and *E. coli*, respectively. Fig. 5a shows a 3D scatter plot of the dry mass versus the circularity and the eccentricity for *S. epidermidis* (blue dots) and *E. coli* (red dots). While the bacterial cells tend to group into two distinguishable clusters, there remains a considerable region where both populations overlap, making difficult their classification. However, in contrast to *E. coli* which is commonly found as single isolated cells after our nebulization procedure, *S. epidermidis* has been found either as single cells or as small clusters (two or more cells) (see Fig. 4b, labels: 2, 4, 6, 7, 9 and 10). The circularity, defined as $\:C=\frac{4\pi\:\times\:Area}{{Perimeter}^{2}}$, provides useful information to distinguish between single cocci from clustered cells (Fig. 5b). Circularity quantifies how closely the shape of an object resembles a perfect circle, with a value $\:C\:=\:1$ as observed in single *S. epidermidis*. To further classify *S. epidermidis* cells, we applied a binormal Gaussian Mixture Model (GMM) to the circularity distribution. Binormal GMM is a probabilistic machine learning algorithm that assumes the data is generated from a mixture of two Gaussian distributions with unknown parameters. This approach allowed us to model the heterogeneity and distinguish between single cocci from clustered cells. Fig. 5c shows the histogram of the circularity (blue bars) for the characterized *S. epidermidis* cells. The total fitted distribution is represented by the solid gray line, while the two individual Gaussian components are shown as green and blue dashed lines for cluster and single cocci, respectively. The single cells distribution has a mean circularity value of $\:{\mu\:}_{1}=0.998$ with a standard deviation $\:{\sigma\:}_{1}=0.020$, while the clusters have a mean circularity value of $\:{\mu\:}_{2}=0.83$ and a standard deviation of $\:{\sigma\:}_{2}=0.098$. Interestingly, the weights of both distributions are comparable, 0.51 and 0.49 for singles and clusters, respectively, indicating that single and clustered cells are present in similar proportions on the nebulized surface. We defined a filter to select single cells as those with a circularity value within the range $\:{\mu\:}_{1}\pm\:{2\sigma\:}_{1}$. Fig. 5d represents the 3D scatter plot of the dry mass versus circularity and eccentricity of the filtered single *S. epidermidis* (blue dots) and the *E. coli* (red dots). After excluding the clusters, two clearly distinguishable regions emerge, allowing for classification of both bacterial strains.

Fig. 5e displays eccentricity versus dry mass for single *S. epidermidis* (blue dots) and *E. coli* (red dots). The eccentricity is defined as $\:\epsilon\:=\sqrt{1-{\left(\frac{b}{a}\right)}^{2}\:}$, where $\:a$ and$\:\:b$ are the semi-major and semi-minor axes, respectively. Eccentricity measures the degree of elongation, with values approaching 1 indicating increasingly elongated shapes, as is characteristic of *E. coli*, that clearly fall around $\:\epsilon\:\:=\:1$. Interestingly, while *S. epidermidis* exhibits a broader distribution, *E. coli* shows a positive correlation between the dry mass and eccentricity. This suggests that bacilli not only accumulate biomass but also elongate during the growth process, resulting in higher eccentricity. Fig. 5f shows the length (red filled circles) and width (red open circles) of *E. coli* bacterial cells versus the dry mass, respectively. While the width is almost constant, the length increases with the dry mass. This is consistent with the known growth mechanisms of rod-shaped bacteria, where cell elongation occurs primarily along the major axis, with almost constant diameter^49–52^. Here, we obtain a linear fit (black dashed line in Fig. 5f) of the cell length as a function of dry mass, given by: $\:L\left(\mu\:m\right)=\:1.193\:+\:0.006\times\:Dry\:Mass\left(fg\right)$. This empirical relationship enables the estimation of the dry mass of this *E. coli* strain simply by measuring cell length using conventional optical microscopy, which is readily available is most laboratories. Furthermore, since elongation is a key feature of the *E. coli* cell cycle, parameters such as eccentricity, as well as the relation between length, width and dry mass, may provide insight into bacterial growth or physiological state^53^.

#### NMS versus DHM

**Fig. 6:**
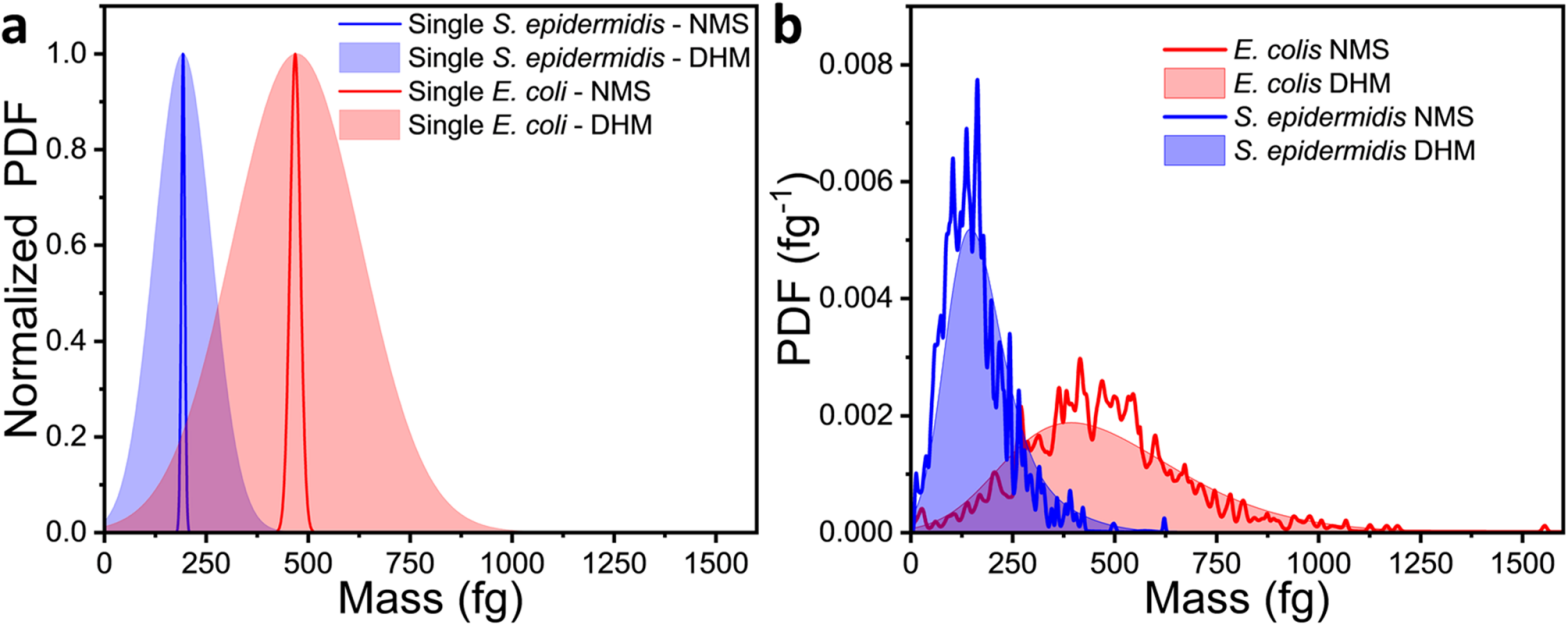
Dry mass distribution of *S. epidermidis* and *E. coli* measured by digital holographic microscopy (DHM) and nanomechanical mass spectrometry (NMS) techniques. (**a**) Normalized dry mass probability density functions (PDF) of representative single cells measured by DHM (filled area) and NMS (solid line) for *S. epidermidis* (blue) and *E. coli* (red). (**b**) Total dry mass PDFs distributions of the studied *S. epidermidis* (blue) and *E. coli* (red) populations obtained by DHM (filled area) and NMS (solid line).

Finally, we compared the dry mass obtained by the high-throughput DHM technique with measurements provided by a state-of-the-art NMS system, which combines ultra-high mass sensitivity with a broad detection mass range^54^. In order to compare both, we have estimated the DHM dry mass uncertainty using standard error propagation techniques given by,

5 $$\:{{\delta\:M}}_{i}=\:\sqrt{{\left(\frac{1}{\alpha\:}{S}_{i}{{\updelta\:}}_{{OPD}}\right)}^{2}+{\left(-\frac{\overline{{{OPD}}_{i}}}{{\alpha\:}^{2}}{S}_{i}{{\updelta\:}}_{\alpha\:}\right)}^{2}+{\left(\frac{\overline{{{OPD}}_{i}}}{\alpha\:}{{\updelta\:}}_{{S}_{i}}\right)}^{2}}\:\:\:$$

where $\:{{\updelta\:}}_{OPD}$ is the associated error of the OPD, which is given by the standard deviation of the whole cell-free background noise (see Fig. 4) for each image, the error associated to the constant $\:\alpha\:$ is $\:{{\updelta\:}}_{\alpha\:}=0.03\times\:{10}^{-4}\:{m}^{3}/kg$, given by the literature^41^, and the surface error $\:{{\updelta\:}}_{{S}_{i}}$ of each bacterial cell was estimated from a 1-pixel morphological dilation/erosion of the mask, which yields $\:{{\updelta\:}}_{{S}_{i}}\approx\:{P}_{i}\bullet\:p$, with $\:{P}_{i}$ the cell perimeter and $\:p$ the pixel size. Fig. 6a shows the dry mass probability density functions (PDFs) of a representative *S. epidermidis* and *E. coli* single cell determined by DHM (filled areas) and NMS (solid lines). We find that the median coefficient of variation (CV) of the dry mass for NMS^7^ is $\:\sim2\%$ and $\:\sim35\%$ for DHM, respectively. The main source of error of DHM method arises from spatial corrugations in the phase, mainly due to optical aberrations introduced by the microscope objective, small misalignments, the liquid medium and the glass cover surface where the bacteria are deposited. The phase at this region is used as a reference to calculate the phase shift induced by the cell ($\:{{\updelta\:}}_{OPD}$). In addition, the phase baseline determines the contour of the image that is regarded as a cell, which affects the estimation of the cells’ area ($\:{{\updelta\:}}_{{S}_{i}}$).

Fig. 6b shows the total PDF mass distribution obtained with the NMS technique^7^ (solid lines) and DHM (filled areas). The mean dry mass of *S. epidermidis* and *E. coli* cells were similar by both techniques, around 180 and 470 fg, respectively. However, we found differences in the mass distributions. Firstly, NMS mass distribution can be discretized into multiple narrow peaks corresponding with most probable masses, whereas DHM mass distribution is almost perfectly smooth due to its lower mass resolution. Although DHM has lower resolution at the single-cell level, it offers several advantages such as it is faster, can operate in liquid environment, and relies on simpler and commercially available equipment.

Finally, we evaluated the DHM dry mass distribution using the Koch & Schaechter model^39,40^, which relates the mass of asynchronously growing cells to the mass at the instant of division while accounting for stochasticity in growth and divisions. The DHM distribution were well fitted by this model, yielding high R-squared values. The model provides an estimated mean division mass of 272 fg for *S. epidermidis* and 751 fg for *E. coli*. The CV of the division mass was around 28% for both strains. These results are in good agreement with our previous work obtained by NMS^7^. Moreover, DHM provides not only dry mass and the division mass but also morphological parameters, such as circularity or eccentricity, which can be highly useful in microbiological field applications.

## Conclusion

In this study, we present a pipeline for the label-free and fast characterization of individual bacterial cells using DHM. By combining polynomial background correction, Gaussian filtering, and adaptive masking, we extract physical parameters such as dry mass, mean division dry mass, eccentricity, and circularity, enabling a comprehensive biophysical and morphological characterization at the single-cell level. The obtained dry mass and division mass distributions of *S. epidermidis* and *E. coli* show broad comparability at the population level with the values obtained from very high mass resolution NMS technique. Although DHM has an inherent measurement noise, it provides significant additional advantages in speed and simplicity, allowing the analysis of hundreds of bacterial cells within minutes, with minimal sample preparation and no labeling. Additionally, DHM simultaneously provides morphological information alongside dry mass. This complementary information enabled us to distinguish individual *S. epidermidis* from clusters ones, and to suggest trends consistent with the expected growth mechanism of *E. coli* cells. Although this study was limited to *S. epidermidis* and *E. coli*, this methodology can be straightforwardly extended to other bacterial types, including filamentous, spiral, or irregular species, to fully capture morphological and dry-mass diversity with no fundamental limitations from a technical perspective.

The potential of DHM as a label-free, user-friendly, and high-throughput technique to obtain both the dry mass and morphological information could be highly valuable for applications such as antibiotic susceptibility testing, studying cell division and aggregation dynamics and rapid detection and classification of bacterial cells. DHM emerges as a powerful tool in microbiology, offering a compelling balance between resolution, accuracy, speed, and the richness of extracted information, opening the door to monitor living bacteria.

## Methods

### Sample preparation

The bacterial strains *E. coli* and *S. epidermidis* were kindly supplied by Dr. Jesús Mingorance from the Microbiology Department at Hospital La Paz (Madrid, Spain) from his research collection. Both strains were used at its stationary phase. Thus, 10 mL of Luria-Bertani (LB) broth (Sigma-Aldrich, USA) were inoculated with 100 µL of a stationary-phase culture and both strains were grown independently overnight at 37 °C under agitation before its use. For *E. coli*, solutions were centrifuged at 2500 rpm for 5 min at 25 °C. For *S. epidermidis*, centrifugation was performed at 4400 rpm during 25 min at 25 °C. After centrifugation, the supernatant was removed and the remaining solutions were resuspended in Milli-Q water. This washing step was repeated three times. Finally, bacteria cells were resuspended in a 50:50 isopropyl alcohol/Milli-Q water solution, and the concentration was adjusted to 10^9^ cells per mL by measuring the optical density at 600 nm using a BioSpectrophotometer (Eppendorf, Germany).

The bacterial cells were inactivated by the sample preparation protocol without compromising their structure. This represent a limitation if the goal is to determine the dry mass of live bacteria in their native medium. The final suspensions were nebulized onto microscope cover glasses using an electrospray ionization system.

### Bacterial deposition using the nanomechanical spectrometer system

Bacterial cells were deposited onto microscope cover glasses (VWR 631–1567, 18 × 18 mm) using a custom-built nanomechanical spectrometer (NMS) prototype equipped with an electrospray ionization system (ESI) source^7^. The ESI source consists of a stainless-steel metal needle (76 μm ID, 152 μm OD, 14.6 cm long, Thermo Fisher Scientific) connected to a high-pressure syringe pump (Base 120 Cetoni Gmbh, Germany) and a high-voltage power supply (HV-7010 Ioner, RAMEN). Bacterial suspensions were nebulized for 30 min at a flow rate of 0.3 µL/min and applying a voltage of 2650 V between the ESI needle and the spectrometer entrance.

The NMS prototype comprised three vacuum chambers^7^. The entrance chamber, maintained at 10 mbar, contains a heated stainless-steel capillary with an inner diameter of 400 μm and a length of 11.4 cm with its temperature set to 150 °C to ensure complete solvent evaporation. After passing through the heated capillary, the charged bacterial cells enter the aerolens system (Fasmatech, Greece), consisting of a long capillary designed to decelerate the particles velocity. Subsequently, the cells are guided to the final chamber at 0.1 mbar, where the microscope cover glass is placed 14 mm downstream from two consecutive skimmers. Under these conditions, bacterial cells were deposited individually onto the microscope cover glass in the absence of residual solvent. After deposition, a drop of LB medium was placed on top of the cover glass containing the deposited cells, and a second cover glass was mounted on top, sandwiching the bacterial cells between both surfaces in liquid medium, suitable for DHM measurements.

### Digital holographic microscopy setup

Quantitative phase images were acquired using a commercial transmission digital holographic microscope (DHM-T2100, Lyncée Tec, Switzerland). The system operates in a transmission off-axis Mach-Zender interferometer configuration, where the object and reference beams reach the CCD camera with an angle *θ*. Measurements were performed with a 666 nm laser source and a 63x/1.30 NA oil immersion objective (HC PL FLUOTAR, Leica), providing a field of view of 83 μm and a lateral resolution of 0.53 μm. The vertical resolution, defined as twice the accuracy, is 2.0 nm. The system employed a CCD camera with an effective pixel size of 90.6 nm at the sample plane. Each hologram was acquired with an exposure time of 0.05 s, integrating 20 frames for a total acquisition time of 1 s. Once the hologram is recorded in the CCD camera, numerical reconstruction is performed using the commercial software Koala (v.8.6.59715.0, Lyncée Tec, https://www.lynceetec.com/koala-acquisition-analysis-software/*)* obtaining the phase image. The first step consists on a Fourier filtering in the spatial frequencies where the twin image and zero diffraction order images are eliminated^55^. Numerical propagation from the hologram plane to the image plane is performed using a scalar diffraction in the Fresnel approximation^37,38^. The introduction of the microscope objective creates phase aberrations that are numerically corrected and largely eliminated following the procedure described by Cuche et al.^38^

### Statistics and reproducibility

#### Biological replicates

The DHM data shown in this work correspond to two independent experiments for each bacterial strain, performed on different days, with fresh ESI nebulization in each session. This study was limited to two bacterial strains and single-preparation samples. In every acquisition, 20 individual holograms were recorded within 1 s, reconstructed using Koala software, and averaged to obtain the phase images. For *S. epidermidis*, 9 and 20 DHM images were acquired in the two independent experiments. For *E. coli*, 9 and 18 DHM images were acquired. After background leveling and Gaussian filtering, this resulted in a total of 179 labeled regions for *S. epidermidis* and 177 for *E. coli*. Further studies should include additional bacterial strains.

#### Background noise reduction

Background noise reduction was performed in two sequential steps. First, the OPD images were corrected using the *BrightnessEqualize* function (Global mode, polynomial order 16, Wolfram Mathematica), which fits and subtracts a 2D polynomial surface. Second, a Gaussian filter (*GaussianFilte*r, 4, Wolfram Mathematica) with a kernel radius of 4 pixels was applied. The Gaussian filter parameter was selected to reduce the noise without modifying the quantitative evaluation of dry mass or the morphological parameters of the bacterial cells. To verify that the polynomial background correction did not bias the quantitative evaluation of dry mass, we used a frozen mask (obtained with a 16th order polynomial and Gaussian filter radius 4) and recalculated the integrated OPD of different bacterial cells using polynomial fits of varying orders (2, 4, 8, 16, 32, 64, and 128). The relative discrepancy found was below 5%, well within the intrinsic measurement uncertainty.

#### Dry mass uncertainty

OPD error was calculated per image as the standard deviation of the OPD over the whole cell-free background regions after background leveling and Gaussian filtering, and used as $\:{{\updelta\:}}_{{OPD}_{i}}$ in error propagation, where the subscript i denotes each independent image. The dry mass uncertainty for each bacterial cell was estimated by standard error propagation (Eq. 5), accounting for contributions from OPD noise, mask area, and refractive index increment. No replicate measurements or manual ground-truth validations were performed. We have computed confidence intervals (CI) using Student’s t-distribution. For *S. epidermidis* the mean error is 36.02% with a 95% CI of (35.42%, 36.62%) and for *E. coli* the mean error is 36.06% with a 95% CI of (35.52%, 36.59%).

#### Masking robustness

After applying background leveling and Gaussian filtering, we performed an adaptive binary masking and labeling in Python using the *scikit-image* library (*skimage.measure.regionprops*). Segmentation robustness was assessed by sweeping threshold scaling (from 1 to 3 times the standard deviation of the whole image, in steps of 0.25), morphological opening and mask-dilation radii (1–3 px), and adaptive block sizes (32, 64, 128 px). The final parameters were selected to maximize cell recall without affecting integrated dry-mass estimates.

#### *E. coli* linear regression

The *E. coli* length-mass linear regression fit yields an intercept of $\:1.19\pm\:0.18\:\mu\:m$ and a slope of $\:\left(5.7\pm\:0.35\right)\bullet\:{10}^{-3}\:\mu\:m/fg$. The residual sum of squares was 79.7, the Pearson correlation coefficient was $\:r=0.77$ and $\:{R}^{2}=0.60$ (*n* = 177, DOF = 175). Although moderate, this $\:{R}^{2}$ reflects intrinsic biological variability of single cell-measurements, but the correlation is consistent with the expected elongation mechanism of *E. coli*.

#### Gaussian mixture model (GMM)

A GMM is an unsupervised machine learning method that models the data distribution as a weighted sum of Gaussians components. In order to validate the model used in this work, 1–5 components were evaluated. We computed the Akaike Information Criterion (AIC) and Bayesian Information Criterion (BIC) that are used to compare different models in order to decide which one is the best. The results obtained shows that while the AIC value decreased slowly with increasing components, approaching a plateau, the BIC value reached a clear minimum at 2 components. Therefore, the 2-components model was selected as the best trade-off, and consistent with SEM observation of two subpopulations (single and clustered cocci). We also evaluated the component overlap. The GMM assigns to each bacterium a probability of belonging to each component. Using a 70% confidence threshold for classification, only the 3.35% of the cocci were classified as ambiguous. With stricter 90% confidence threshold, the fraction of ambiguous cases increases to 11.73%. The segmentation and GMM classification were implemented as a proof-of-concept method and no validation against manually annotated ground truth was performed.

#### Comparison of DHM and NMS dry mass distributions

To compare the dry mass distribution obtained with DHM and NMS (Fig. 6b) we computed the Earth Mover’s Distance (EMD) between the discrete dry mass values obtained from the DHM and the mean mass values calculated from each PDF from the NMS experiments. In addition, 10,000 bootstrap resample were generated from the PDFs shown in the manuscript. The EMD values showed good concordance between both techniques: 21 fg from *S. epidermidis* and 27 fg for *E. coli* from the bootstrap analysis, and 24 fg for *S. epidermidis* and 37 fg for *E. coli* from the discrete dry mass mean values. These results, together with the visual agreement of the distributions, provide strong evidence that the two techniques yield comparable dry mass distributions.

#### Koch & schaechter model

We evaluated the mean mass and coefficient of variation (CV) at the instant of division using the classical Koch & Schaechter model, which relates the steady-state size distribution of asynchronously growing cells to the division mass, and have into account the effect of stochasticity in cell dynamics^7,39,40^. In this model, the probability density of the dry mass $\:m$ is:

$$\:\theta\:\left(m\right)=\frac{A}{{m}^{2}}\underset{m}{\overset{2m}{\int\:}}g\left(x\right)\:dx$$

where $\:g\left(x\right)$ is the probability density of the division mass and $\:A$ is a normalization constant. Assuming a normal distribution:

$$\:g\left(x\right)=\frac{1}{\sqrt{2\pi\:}{\sigma\:}_{div}}{e}^{-\frac{{\left(x-{\mu\:}_{div}\right)}^{2}}{2{\sigma\:}_{div}^{2}}}$$

this leads to:

$$\:\theta\:\left(m\right)=\frac{A}{2{m}^{2}}\left(\text{erf}\left(\frac{\left({\mu\:}_{div}-m\right)}{\sqrt{2}{\sigma\:}_{div}}\right)-\text{erf}\left(\frac{\left({\mu\:}_{div}-2m\right)}{\sqrt{2}{\sigma\:}_{div}}\right)\right)$$

This function was fitted to DHM dry mass distributions obtaining the mean division mass, $\:{\mu\:}_{div}$, its dispersion, $\:{\sigma\:}_{div}$, and the coefficient of variation, (CV). For *S. epidermidis* DHM yielded $\:{\mu\:}_{div}=272\:fg$ and $\:{\sigma\:}_{div}=77\:fg$ with CV = 28% ($\:{R}^{2}=0.97$) while NMS gave $\:{\mu\:}_{div}=243\:fg$ and $\:{\sigma\:}_{div}=76\:fg$ with CV = 31% ($\:{R}^{2}=0.97$). For *E. coli*, DHM yielded $\:{\mu\:}_{div}=751\:fg$ and $\:{\sigma\:}_{div}=213\:fg$ with CV = 28% ($\:{R}^{2}=0.96$), and NMS gave $\:{\mu\:}_{div}=758\:fg$ and $\:{\sigma\:}_{div}=210\:fg$ with CV = 28% ($\:{R}^{2}=0.95$). The results demonstrate that both techniques are in good agreement.

## Data Availability

The datasets generated and/or analyzed during the current study are available from the corresponding author under reasonable request.
